# Risk factors for distant recurrence in patients with rectal cancer supporting selection for total neoadjuvant therapy

**DOI:** 10.2340/ao.v65.45169

**Published:** 2026-04-17

**Authors:** Anna Baech Slipsager, Rana Bahij, Heidi Søgaard Christensen, Laura Vittrup Diness, Birgitte Mayland Havelund, Anne Ramlov, Signe Korsgaard Skriver, Joanna Eliza Szpejewska, Olga Tcacenco, Christian Thomsen, Laurids Østergaard Poulsen

**Affiliations:** a Department of Oncology, Aalborg University Hospital, Aalborg, Denmark; b Clinical Cancer Research Center, Aalborg University Hospital, Aalborg, Denmark; c Department of Clinical Medicine, Aalborg University, Aalborg, Denmark; d Department of Oncology, Odense University Hospital, Odense, Denmark; e Center for Clinical Data Science, Aalborg University, Aalborg, Denmark; f Department of Oncology, Copenhagen University Hospital – Herlev and Gentofte, Herlev, Denmark; g Department of Oncology, University Hospital of Southern Denmark, Lillebaelt Hospital, Vejle, Denmark; h Department of Oncology, Aarhus University Hospital, Skejby, Denmark; i Department of Oncology, Rigshospitalet, Copenhagen University Hospital, Denmark; j Department of Oncology, Zealand University Hospital, Roskilde, Denmark; k Department of Radiology, Aalborg University Hospital, Aalborg, Denmark; l Department of Pathology, Aalborg University Hospital, Aalborg, Denmark

**Keywords:** Rectal neoplasms, neoadjuvant therapy, radiotherapy, risk factors

## Abstract

**Background and purpose:**

In locally advanced rectal cancer, distant recurrence remains a major challenge. Total neoadjuvant therapy (TNT) has been implemented to reduce this risk. Identification of risk factors for distant recurrence to support selection of TNT is crucial to avoid excessive treatment. This study aimed to identify magnetic resonance imaging (MRI) features predictive of distant recurrence in patients with rectal cancer.

**Material and methods:**

In this national retrospective cohort study, all patients were diagnosed with non-metastatic rectal cancer between 2016 and 2020 and received neoadjuvant radiotherapy followed by surgery. MRI features from the diagnostic report, including cT-stage, cN-stage, clinical extramural vascular invasion (cEMVI), distance to the mesorectal fascia (MRF), and tumor deposit, were registered. The primary endpoint was distant recurrence.

**Results:**

A total of 648 patients were included in the study. In multivariate analysis using multiple imputation, risk factors significantly associated with distant recurrence were cT3c/cT3d/cT4-stage (hazard ratio [HR]: 1.8), cEMVI positive tumors (HR: 2.05), and cT3-stage tumors with a distance to MRF of ≤ 2 mm (HR: 1.51) compared with patients with cT1/cT2/cT3/cT3a/cT3b-stage, cEMVI negative tumors, and cT3-stage tumors with a distance to MRF > 2 mm, respectively. The cN-stage was not significantly associated with distant recurrence. Due to 67% missing data on tumor deposit status, the results regarding this feature cannot be considered conclusive.

**Interpretation:**

In this study, cT-stage, cEMVI status, and distance to MRF are prognostic features for risk stratification for patients receiving neoadjuvant radiotherapy. These results may be used for selecting patients for future treatment strategies such as TNT.

## Introduction

Over recent decades, improvement of diagnostics and treatment for locally advanced rectal cancer (LARC) have significantly reduced the risk of local recurrence to approximately 5% [1–3]. In contrast, the incidence of distant recurrence have until recently remained high at 20–30% [2, 4, 5]. Total neoadjuvant therapy (TNT) strategies have been implemented due to the results from the RAPIDO and PRODIGE 23 trials, that demonstrated a 25–31% relative reduction in the risk of distant recurrence [6, 7]. TNT consists of neoadjuvant radiotherapy and chemotherapy. Some regimes also include postoperative adjuvant chemotherapy [8, 9].

Although both the RAPIDO and the PRODIGE 23 trial enrolled LARC patients with a high risk of recurrence, the inclusion criteria had some differences. In PRODIGE 23, high-risk features included cT3-stage rectal cancer recommended neoadjuvant chemoradiotherapy by a multidisciplinary tumor board or cT4-stage rectal cancer [9]. The RAPIDO trial defined high-risk patients based on cT4a/cT4b-stage, clinical extramural vascular invasion (cEMVI), cN2, involved mesorectal fascia (MRF), or enlarged lateral lymph nodes [1]. Additional studies evaluating alternative TNT regimens and inclusion criteria have been published, resulting in several viable options now recognized in national and international guidelines [8, 10]. The ESMO recommendations adopt the RAPIDO risk factors for selecting patients with high-risk LARC for TNT. Furthermore, the recommendations add tumor deposit, due to association with shorter disease-free survival, as a possible high-risk factor when properly validated by future studies [11]. However, while TNT reduces the risk of recurrence, it carries the risk of overtreatment and considerable treatment-related side effects compared to standard treatment with long-course chemoradiotherapy and short-course radiotherapy. TNT treatment has been associated with higher risk of serious adverse events such as diarrhea, nausea, neutropenia, and fatigue compared to standard of care [12].

Identification of predictive risk factors for distant recurrence is essential to guide optimal neoadjuvant treatment selection in patients with LARC. This study aims to identify magnetic resonance imaging (MRI) features predictive of distant recurrence in patients with rectal cancer.

## Materials and methods

This national study was reported in accordance with the STROBE guidelines for cohort studies [13]. The cohort was retrieved from the Danish Colorectal Cancer Group’s (DCCG) database collecting all consecutive Danish patients treated with neoadjuvant chemoradiotherapy followed by surgery from 2016 to 2020. The database records clinical information of rectal cancer patients in Denmark with a median overall accuracy of 95% [14]. Inclusion criteria were diagnosis with a non-metastatic rectal cancer from 1^st^ of January 2016 to 31^st^ of December 2020, tumor distance to the anal verge of 15 centimeter or less, age between 18 and 80 years, a diagnostic MRI-scan, and treatment with curative-intended radiotherapy followed by surgery. Patients were excluded if they had synchronous colorectal cancer or were not Danish residents to ensure complete access to medical records during follow-up.

The DCCG database does not contain sufficient information regarding the diagnostic MRI-scan, oncological treatment, and follow-up data such as overall survival as well as local- and distant recurrence. These details were obtained from the patients’ electronic health records. Using the REDCap platform [15, 16], descriptive data and outcomes from the DCCG database and patients’ electronic health records were registered. All data from the patients’ electronic health records were reviewed and registered by expert gastrointestinal oncologists. In the institution-specific description of each diagnostic MRI-scan, patients’ cT-stage, cN-stage, cEMVI, distance to MRF, tumor deposit, distance to anal verge, and tumor depth were noted and entered in REDCap. The distance to MRF was recorded only for cT3 tumors. When available, it was documented as an exact measurement in millimeters; otherwise, it was noted as either ≤ 2 or > 2 mm. This categorization was introduced following the 2018 Danish national guidelines for neoadjuvant oncological treatment, which recommended therapy for patients with an MRF distance of ≤ 2 mm. Consequently, some patients have a recorded category (≤ 2 mm or > 2 mm) rather than a precise numerical measurement. The maximum tumor depth beyond muscularis propria was only recorded for cT3 tumors. When available, it was documented as an exact measurement in millimeters; otherwise, it was noted as either ≤ 5 mm or > 5 mm. Patients with a cT3-stage tumor with a tumor depth of ≤ 5 mm were defined as cT3a/b, whereas those with a cT3-stage tumor with a tumor depth of > 5 mm were defined as cT3c/d. Patients without a registered tumor depth were defined as cT3-stage.

Where multidisciplinary team meetings resulted in modification of the MRI features, the final clinical staging was registered. If cEMVI or tumor deposit was neither noted in the diagnostic scan nor mentioned in the multidisciplinary meeting minute, it was registered as ‘not documented in the diagnostic MRI report’. Treatment details such as the dose of neoadjuvant radiotherapy, type of neoadjuvant chemotherapy and adjuvant chemotherapy, and completion of therapy were noted.

Patients were followed from date of surgery until local recurrence, distant recurrence, simultaneously local and distant recurrence, death, or end of follow up the 31st of December 2024, whichever occurred first. The primary endpoint was distant recurrence, and the secondary endpoints were overall survival and local recurrence. The date of local and/or distant recurrence was defined as either the date when the local and/or distant recurrence was first detected radiologically or as the date when patient’s symptoms led to an investigation that subsequently confirmed the recurrence.

### Statistical analysis

Baseline demographic and clinical variables were summarized using appropriate descriptive statistics for both the whole patient cohort and stratified on missingness in cEMVI and tumor deposit. Continuous data were reported as medians with minimum and maximum value and categorical data as counts and percentages. The extent of missing documentation of cEMVI and tumor deposit in the diagnostic MRI report was assessed per year of diagnosis. Characteristics were compared between patients with and without missing cEMVI or tumor deposit using Fisher’s exact test for categorical variables with expected cell counts less than five, or Pearson chi-square test otherwise. Age was compared using Wilcoxon rank sum test. The relationship between distance to cEMVI status and distance to MRF was summarized using a contingency table with percentages calculated for each category of cEMVI status.

Overall survival was estimated using the Kaplan–Meier method. Cumulative incidence functions were calculated for local recurrence and distant recurrence, treating each event as a competing risk for the other, with death considered as an additional competing event. Differences in cumulative incidence for distant recurrence were assessed using Gray’s test for cT-stage, cN-stage, cEMVI, distance to MRF, and tumor deposit. The association between MRI features and risk of distant recurrence was examined using cause-specific Cox proportional hazards regression, treating death and local recurrence as competing events. Univariate Cox proportional hazards regressions were performed for cT-stage, cEMVI, and distance to MRF, followed by multivariate models adjusting for cN-stage, type of chemotherapy, and type of radiotherapy. Adjustments were made for the tumor depth for cT3 tumors in the multivariate analysis for distance to MRF and for cT-stage in analysis for cEMVI. Both complete-case analyses and multiple imputation for missing data were conducted. cTx-stage and cNx-stage were considered as missing data. Missing covariate data were imputed using multiple imputation by chained equations to generate 20 datasets with the imputation model including age, sex, cT-stage, cN-stage, cEMVI, distance to MRF, tumor deposit, tumor depth, and distance to anal verge as well as time to distant recurrence and death. Continuous variables were imputed using predictive mean matching, categorical variables using (multinomial) logistic regression. Estimates from the imputed datasets were pooled according to Rubin’s rules to obtain hazard ratios (HRs), 95% confidence intervals (CIs), and *p*-values. *P*-values < 0.05 were considered statistically significant.

Analyses were performed in R (version 4.5.1) using the *tidycmprsk* package (version 1.1.0) for cumulative incidence functions and *survival* package (version 3.8-3) for Kaplan–Meier curve for overall survival and Cox proportional hazards regressions [17]. For multiple imputation analyses, the *mice* package was used (version 3.19.0) [18].

## Results

**Figure 1:**
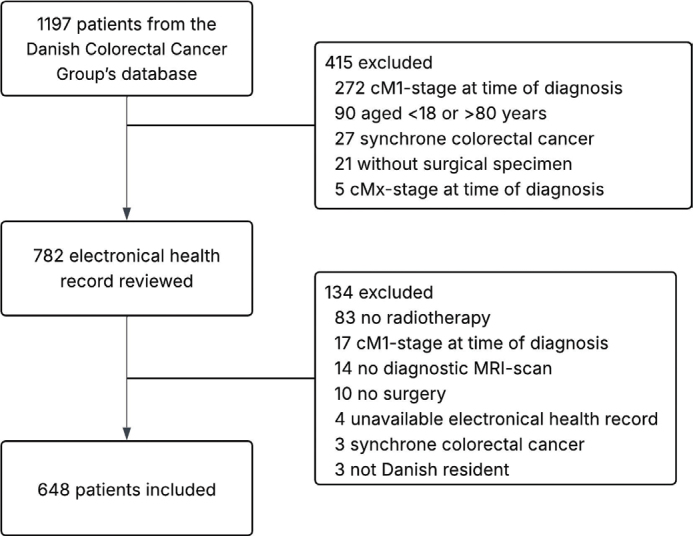
Flowchart.

**Figure 2:**
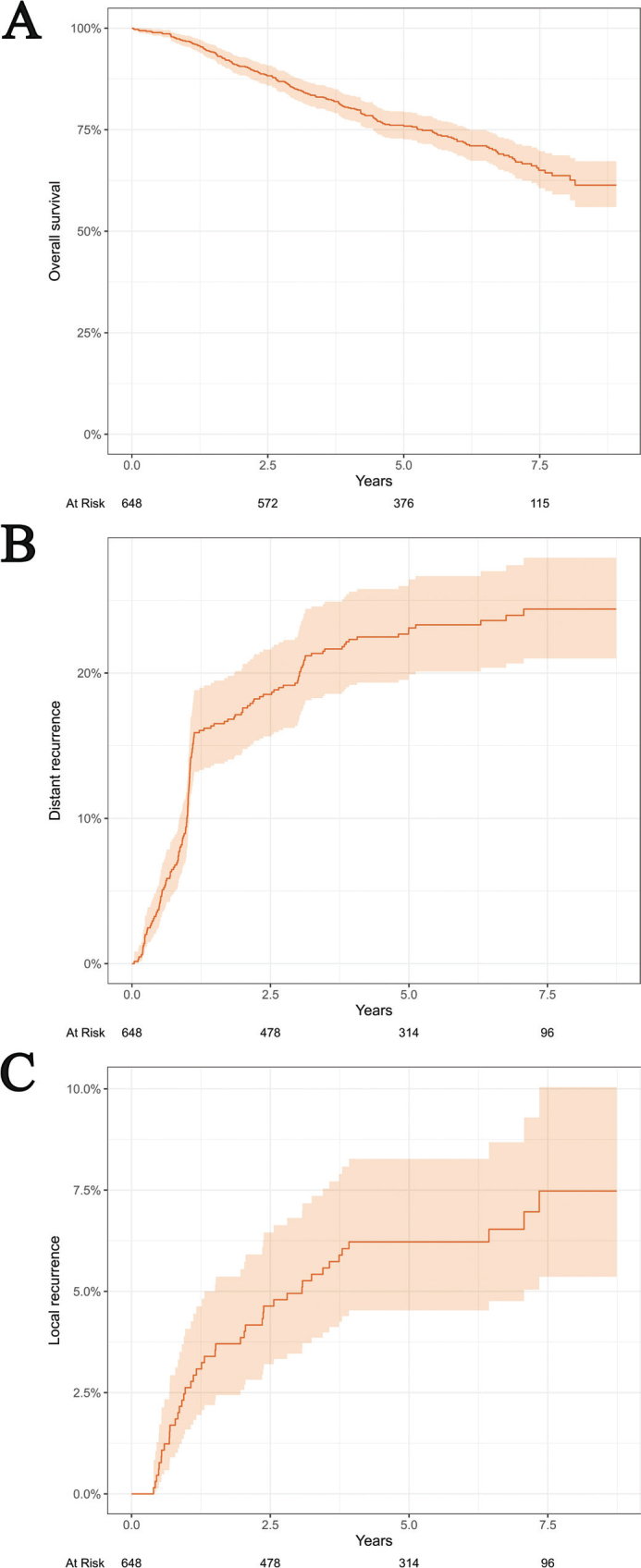
Kaplan–Meier estimate of overall survival (A) and cumulative incidence of distant recurrence (B) and local recurrence (C).

A total of 648 patients were included in this study (Figure 1). Patient characteristics are shown in Table 1. All patients except two finished their planned radiotherapy regime. A total of 192 patients died during a median follow-up time of 6.47 years (95% CI: 6.21–6.67). The 5-year overall survival was 76% (95% CI: 73–79) (Figure 2). The 5-year cumulative incidence was 6% for local recurrence and 23% for distant recurrence (Figure 2).

**Table 1:** Patients’ characteristics.

Included patients (*n* = 648)	
**Sex**	
Female	259 (40.0%)
Male	389 (60.0%)
**Age**	
Mean (minimum to maximum)	64 (21–80)
**Distance to anal verge** ^1^	
0–5 cm	307 (47.4%)
>5–10 cm	297 (45.8%)
>10–15 cm	40 (6.2%)
Not documented in the diagnostic MRI report	4 (0.6%)
**cT-stage** ^1^	
cT1–T2	27 (4.2%)
cT3	464 (71.6%)
cT4	155 (23.9%)
cTx	2 (0.3%)
**cN-stage** ^1^	
cN0	145 (22.4%)
cN1	197 (30.4%)
cN2	295 (45.5%)
cNx	11 (1.7%)
**cEMVI** ^1^	
Yes	261 (40.3%)
No	159 (24.5%)
Not documented in the diagnostic MRI report	228 (35.2%)
**Distance to MRF for cT3 tumors** ^1^	
≤2 mm	284 (43.8%)
>2 mm	162 (25.0%)
Not documented in the diagnostic MRI report	18 (2.8%)
cT1, cT2, or cT4 tumors	184 (28.4%)
**Tumor deposit** ^1^	
Yes	48 (7.4%)
No	166 (25.6%)
Not documented in the diagnostic MRI report	434 (67.0%)
**Tumor depth for cT3 tumors** ^1,3^	
≤5 mm (cT3a and cT3b)	226 (34.9%)
>5 mm (cT3c and cT3d)	202 (31.2%)
Not documented in the diagnostic MRI report (cT3)	36 (5.6%)
cT1, cT2, or cT4 tumors	184 (28.4%)
**Neoadjuvant radiotherapy**	
25 Gy in 5 fractions	100 (15.4%)
45–52 Gy in 25–28 fractions	537 (82.9%)
60 Gy in 30 fractions	11 (1.7%)
**Neoadjuvant chemotherapy**	
No	113 (17.4%)
Yes	535 (82.6%)
5-FU concomitant with radiotherapy	498/535 (93.1%)
5-FU + oxaliplatin	12/535 (2.2%)
Other combinations of chemotherapy	25/535 (4.7%)
**Microscopically radical resection** ^2^	
Yes	569 (87.8%)
No	70 (10.8%)
Not documented in the histopathological report	9 (1.4%)
**Adjuvant chemotherapy**	
No	627 (96.8%)
Yes	21 (3.2%)
5-FU	8/21 (38.1%)
5-FU + oxaliplatin	13/21 (61.9%)

cEMVI: clinical extramural vascular invasion; MRF: mesorectal fascia.

1 These variables are based on the diagnostic MRI report.

2 This variable is based on the histopathological report of the surgical specimens.

3 Tumor depth beyond muscularis propria.

### Evaluation of potential risk factors to support selection for TNT

A total of 36 patients with cT3-stage tumors had no reported tumor depth in the MRI report. In multivariate complete-case analysis for distant recurrence, patients with cT3c/cT3d/cT4-stage had a HR of 1.76 (95% CI: 1.20–2.58, *p*-value: 0.004) compared to patients with cT1/cT2/cT3/cT3a/cT3b-stage (Table 2). The results were non-significant for differences in the cumulative incidence of distant recurrence, when comparing cT1/cT2/cT3-stage with T4-stage (Supplementary Material, Figure A). A total of 261 patients had cEMVI-positive rectal cancer and 159 had cEMVI-negative rectal cancer (Table 1). In multivariate complete-case analysis, the risk of a distant recurrence for patients with cEMVI-positive rectal cancer was twice as high compared to patients with cEMVI-negative rectal cancer (HR of 2.09 [95% CI: 1.30–3.36, *p*-value: 0.002]) (Table 2). When using multiple imputation in uni- and multivariate analyses regarding cT-stage and cEMVI status, the results remained close to the results from the complete-case analyses (Table 2).

**Table 2:** Uni- and multivariate Cox-regression for distant recurrence: cT-stage, cEMVI, and distance to MRF.

Characteristics	Univariate			Multivariate		
	HR	95% CI	*p*	HR	95% CI	*p*
**Complete-cases**						
cT-stage^1^						
T1-T2-T3-T3a-T3b	Reference			Reference		
T3c-T3d-T4	1.65	1.14, 2.37	**0.007**	1.76	1.20, 2.58	**0.004**
cEMVI status^2^						
No	Reference			Reference		
Yes	2.02	1.29, 3.14	**0.002**	2.09	1.30, 3.36	**0.002**
Distance to MRF^3^						
> 2 mm	Reference			Reference		
≤ 2 mm	1.47	0.98, 2.21	0.062	1.39	0.91, 2.12	0.13
**Multiple imputation**						
cT-stage						
T1-T2-T3-T3a-T3b	Reference			Reference		
T3c-T3d-T4	1.66	1.15, 2.4	**0.008**	1.8	1.23, 2.63	**0.003**
cEMVI status						
No	Reference			Reference		
Yes	2.01	1.26, 3.21	**0.004**	2.05	1.23, 3.42	**0.007**
Distance to MRF						
> 2 mm	Reference			Reference		
≤ 2 mm	1.49	0.99, 2.24	0.058	1.51	1.0, 2.29	**0.049**

CI: confidence interval; HR: hazard ratio; cEMVI: clinical extramural venous invasion; MRF: mesorectal fascia.

Number of patients removed in complete-case analysis due to missing documentation in the diagnostic MRI report:

1 univariate: 0 patients, multivariate: 14 patients,

2 univariate: 228 patients, multivariate: 243 patients,

3 univariate: 18 patients, multivariate: 61 patients.

In multivariate analysis using multiple imputation, cT3-stage tumors with a distance to MRF of ≤ 2 mm were associated with an increased risk of distant recurrence when compared with cT3-stage tumors with a distance > 2 mm (HR 1.51, 95% CI: 1.00–2.29, *p*-value: 0.049) (Table 2). The distribution of distance to MRF (≤ 2 mm vs. > 2 mm) was similar among patients with cEMVI-positive and cEMVI-negative rectal cancer (Supplementary Material, Table A).

**Figure 3:**
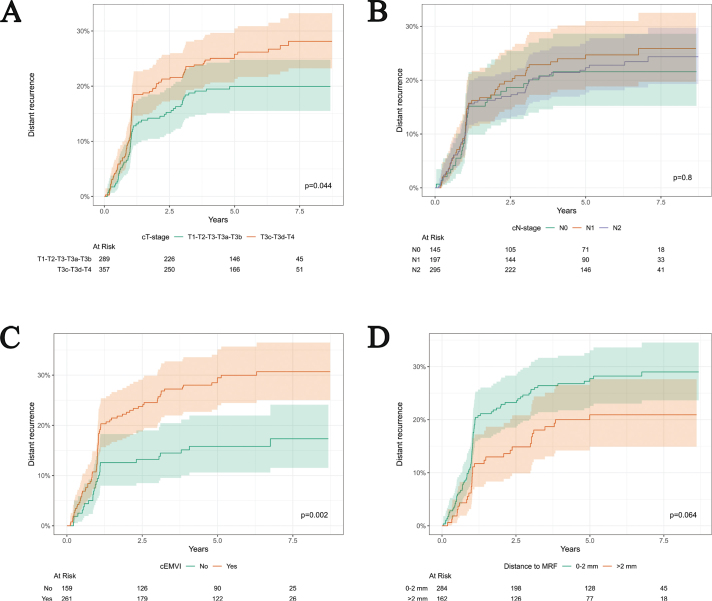
Cumulative incidence of distant recurrence by cT-stage, cN-stage, cEMVI status, and distance to MRF for patients with rectal cancer. (A) cT-stage association with distant recurrence. Two patients with cTx-stage documented in the diagnostic MRI report were excluded in analysis. (B) cN-stage association with distant recurrence. A total of 11 patients with cNx-stage documented in the diagnostic MRI report were excluded in analysis. (C) cEMVI (clinical extramural vascular invasion) association with distant recurrence. A total of 228 patients without cEMVI-status documented in the diagnostic MRI report were excluded in analysis. (D) Distance to MRF (mesorectal fascia) association with distant recurrence for patients with cT3 tumors. A total of 18 patients without distance to MRF documented in the diagnostic MRI report were excluded in analysis. cEMVI: clinical extramural venous invasion; MRF: mesorectal fascia.

No significant differences in the cumulative incidence of distant recurrence were observed according to cN-stage (Figure 3). Results from the tumor deposit analysis should be interpreted with caution and cannot be considered conclusive due to 67% missing data (Supplementary Material, Figure B).

### Missing information in the diagnostic MRI report

For complete-case Cox regressions and cumulative incidence of distant recurrence due to missing information in the diagnostic MRI report, two patients were excluded for cT stage, 11 for cN stage, 18 for distance to MRF, 228 for cEMVI, and 436 for tumor deposits. Patients with and without missing information regarding cEMVI and tumor deposit were compared based on baseline characteristics and outcome (Supplementary Material, Table B & C). Patients with a reported cEMVI status differed from patients missing cEMVI status regarding distance to anal verge, cN-stage, tumor deposit status, and neoadjuvant radiotherapy (Supplementary Material, Table B). Patients with a reported tumor deposit status differed from patients with missing tumor deposit status regarding cN-stage, cEMVI status, neoadjuvant radiotherapy, and neoadjuvant chemotherapy (Supplementary Material, Table C). There was similar 5-year risk of distant recurrence in both groups. When examining the distribution of missing information regarding cEMVI, there was no clear improvement in the reporting from 2016 to 2020 (Supplementary Material, Figure C). For tumor deposit, the highest percentage of missing information was observed in 2016 with a steady rise in reporting until 2019–2020 (Supplementary Material, Figure D).

## Discussion and conclusion

Identifying risk factors is essential for selecting of patients with rectal cancer for TNT, ensuring that those with a high risk of distant recurrence receive treatment while minimizing overtreatment. The study’s results indicate that cT3c/cT3d/cT4-stage tumors, cEMVI positive tumors, and cT3-stage tumors located ≤ 2 mm from MRF are significantly associated with increased risk of distant recurrence.

This study highlights the importance of T3 subclassification. There was no significant association between cT4-stage and distant recurrence when compared with cT1-cT3-stages. However, patients with a cT3c/cT3d/cT4-stage have an increased risk of distant recurrence compared to patients with cT1/cT2/cT3/cT3a/cT3b-stage. This could be explained by the difference in prognosis for cT3-tumors, which can be subclassified from cT3a to cT3d depending on the depth of invasion beyond the muscularis propria [19]. cT3c- and cT3d-stage are associated with a significantly worse overall survival and disease-free survival compared to patients with cT3a- and cT3b-stage [20]. Placing all cT3-stage tumors in the same category might weaken the association with distant recurrence.

cEMVI-positive tumors have a significantly higher risk of distant recurrence compared to patients with cEMVI-negative tumors. However, the assessment of cEMVI relies on the reporting of the radiologist, and data were collected between 2016 and 2020, when cEMVI was not yet a decisive parameter in treatment planning in Denmark. Consequently, cEMVI was inconsistently documented. We therefore considered patients with unknown cEMVI status to represent a mixed cEMVI-positive and cEMVI-negative group. The missing cEMVI data were not systematically related to the year of diagnosis (Supplementary Material, Figure B). When incorporated into the distant recurrence analysis, patients with unknown cEMVI status showed recurrence rates intermediate between the cEMVI-positive and cEMVI-negative groups, supporting the assumption of a mixed profile within this population (Supplementary Material, Figure D).

Other studies have confirmed the predictive value of cEMVI-positive rectal cancer in relation to distant recurrence. Paul et al. demonstrated that cEMVI is an independent risk factor for distant recurrence in LARC. The study included a small cohort of 92 patients with MRI-confirmed cEMVI-positive rectal cancer matched to 92 patients with cEMVI-negative rectal cancer [21]. Smith et al. reported that cEMVI-positive rectal cancer was associated with compromised relapse-free survival in a univariate analysis of 121 rectal and sigmoid cancer patients, but not in multivariate analysis. The latter was explained by a low number of participants [22]. This study, with its large cohort of 420 patients with a registered cEMVI status, shows that an adequate sample size is crucial to demonstrate a robust association between cEMVI-positivity and distant recurrence. This is further supported by a meta-analysis encompassing six studies with a total of 1,262 rectal cancer patients, reporting that patients with cEMVI-positive rectal cancer had more than a threefold higher risk of distant recurrence compared with patients with cEMVI-negative rectal cancer [23].

The distance to MRF assessed by MRI is known to have high sensitivity and specificity [24, 25]. It is established that a distance to MRF above 1 mm is associated with improved prognostic outcome [26]. This criterion was adopted in the 2024 DCCG guidelines for neoadjuvant oncological treatment. Previously, a cutoff of ≤ 2 mm was used to determine eligibility for neoadjuvant (chemo)radiotherapy. The study cohort was treated according to these earlier recommendations, which makes it necessary to interpret the results in the context of the former guidelines. The results indicate that cT3-stage tumors with a distance to MRF of ≤ 2 mm is associated with an increased risk of distant recurrence compared with cT3-stage tumors with a distance to MRF > 2 mm (Table 2). For both univariate analyses, a distance to MRF of ≤ 2 mm showed a trend toward association with distant recurrence compared to a distance to MRF > 2 mm (Table 2 and Figure 3). In a retrospective single-center study by Zhang et al., both cEMVI-positive rectal cancer and rectal cancer with ≤ 1 mm distance to MRF were associated with a greater risk of distant recurrence in both uni- and multivariate analysis [27]. Therefore, a distance to MRF of ≤ 1 mm could still be a possible risk factor for selecting TNT for patients with high-risk rectal cancer.

Relevance of cN-stage have been widely discussed due to a lack of accuracy, which is well documented [26]. The American Society of Clinical Oncology’s recommendations supporting TNT has not included this cN2-stage as a risk factor [28]. This is due to a large Dutch study on 2,178 rectal cancer with patients, where cN0-stage and cN1/cN2-stage was compared to pN0- and pN1/pN2-stage for patients who had not received any neoadjuvant oncological treatment. The sensitivity and positive predictive value for cN1/cN2-stage were 38% and 56%, respectively [29]. The study’s results show that cN2-stage is not associated with distant recurrence. By summarizing previous and current evidence, cN2-stage disease is likely to carry the greatest potential for overtreatment if considered as a single high-risk MRI feature.

This study has some limitations. It is a retrospective design, some data are missing due to incomplete descriptions of MRI features in the diagnostic MRI reports, and no radiologists were involved in the collection of MRI features. It is unknown if the missing data are random or systematic. Missing data could be systematic if radiologists are more likely to report positive MRI features rather than negative. However, assessment of positive or negative MRI features did not have any clinical consequence from 2016 to 2020. This could lead to a random lack of reporting disregarding positive or negative MRI features. Nevertheless, the study also has several important strengths. It is based on real-world data from a large national cohort of 648 patients. All patients are diagnosed with rectal cancer and treated with neoadjuvant curatively intended radiotherapy followed by surgery. Both internal and external validity are high, as expert gastrointestinal oncologists directly extracted and reported the data from patients’ electronic health records within a national cohort.

TNT improves prognosis in patients with rectal cancer with a high risk of recurrence. However, the potential for excessive treatment and consequent increased risk of serious adverse events underscore the need for evidence-based risk stratification to guide patient selection for TNT. In conclusion, based on a consecutive national cohort with real-world data, this study demonstrates that cT-stage, cEMVI status, and distance to MRF are prognostic features for risk stratification for patients receiving neoadjuvant radiotherapy. These findings may be used for selecting patients for future treatment strategies such as TNT.

## Supplementary Material



## Data Availability

Data cannot be made available due to the Danish General Data Protection Regulation.
