# Supplementary material for: Risk factors for distant recurrence in patients with rectal cancer supporting selection for total neoadjuvant therapy
Source: Acta Oncol. 2026 Apr 17;65:45169. doi: 10.2340/ao.v65.45169 (PMC13097092; doi:10.2340/ao.v65.45169)
Supplement: Supplementary file 1 [file AO-65-45169-s1.pdf]

Supplementary material has been published as submitted. It has not been copyedited, or typeset by Acta Oncologica

Risk Factors for Distant Recurrence in Patients with Rectal Cancer Supporting Selection for Total Neoadjuvant Therapy

Supplementary Material

Abbreviations
cEMVI = clinical extramural vascular invasion
MRF = mesorectal fascia

Table A) Distribution of distance to MRF within levels of cEMVI.

	cEMVI			
	No	Yes	Missing	Total
MRF				
0-2 mm	70 (44%)	118 (45%)	96 (42%)	284 (44%)
>2 mm	40 (25%)	66 (25%)	56 (25%)	162 (25%)
Missing	49 (31%)	77 (30%)	76 (33%)	202 (31%)

Table B) Patient characteristics for patients with or without cEMVI status

**Characteristic**	**Missing cEMVI status**	**Reported cEMVI status**	**p-value**
Number of patients	228	420	
Sex			0.6
Female	88 (39%)	171 (41%)	
Male	140 (61%)	249 (59%)	
Age	64 (25 to 80)	64 (21 to 80)	0.9
Distance to anal verge*			**0.010**
0-5 cm	126 (55%)	181 (43%)	
>5-10 cm	91 (40%)	206 (49%)	
>10-15 cm	9 (3.9%)	31 (7.4%)	
Missing	2 (0.9%)	2 (0.5%)	
cT-stage*			0.9

Characteristic	Missing cEMVI status	Reported cEMVI status	p-value
T1-T2	11 (4.8%)	16 (3.8%)	
T3	162 (71%)	302 (72%)	
T4	54 (24%)	101 (24%)	
Tx	1 (0.4%)	1 (0.2%)	
cN-stage*			**<0.001**
N0	72 (32%)	73 (17%)	
N1	71 (31%)	126 (30%)	
N2	80 (35%)	215 (51%)	
Nx	5 (2.2%)	6 (1.4%)	
Distance to MRF for cT3 tumors*			0.3
0-2 mm	96 (42%)	188 (45%)	
>2 mm	56 (25%)	106 (25%)	
Irrelevant (not cT3 tumor)	66 (29%)	118 (28%)	
Missing	10 (4.4%)	8 (1.9%)	
Tumor deposit*			**<0.001**
Yes	4 (1.8%)	44 (10%)	
No	46 (20%)	120 (29%)	
Missing	178 (78%)	256 (61%)	
Neoadjuvant radiotherapy			**<0.001**
25Gyx5F	17 (7.5%)	83 (20%)	
45-52Gyx25-28F	206 (90%)	331 (79%)	
Andet	5 (2.2%)	6 (1.4%)	
Neoadjuvant chemotherapy			0.14
No	33 (14%)	80 (19%)	
Yes	195 (86%)	340 (81%)	
Neoadjuvant chemotherapy type			0.4
5-FU concomitant with radiotherapy	183 (80%)	315 (75%)	
Other combinations of chemotherapy	9 (3.9%)	16 (3.8%)	
None	33 (14%)	80 (19%)	
Missing	3 (1.3%)	9 (2.1%)	
Micro radicality			0.088
No	28 (12%)	42 (10%)	
Yes	194 (85%)	375 (89%)	
Missing	6 (2.6%)	3 (0.7%)	
Adjuvant chemotherapy			0.093
No	217 (95%)	410 (98%)	
Yes	11 (4.8%)	10 (2.4%)	
Adjuvant chemotherapy type			0.2
5-FU	4 (1.8%)	4 (1.0%)	
5-FU + oxaliplatin	7 (3.1%)	6 (1.4%)	
None	217 (95%)	410 (98%)	

N (%), Mean (minimum to maximum)

5-year distant recurrence probability: Missing: 0.21 (95% CI: 0.16, 0.26), Non-missing: 0.24 (95% CI: 0.2, 0.29)

Table C: Patient characteristics for patients with or without tumor deposit status

Characteristic	Missing tumor deposit status	Reported tumor deposit status	p-value
Number of patients	434	214	
Sex			0.2
Female	181 (42%)	78 (36%)	
Male	253 (58%)	136 (64%)	
Age	64 (21 to 80)	65 (22 to 80)	0.4
Distance to anal verge*			0.2
0-5 cm	201 (46%)	106 (50%)	
>5-10 cm	206 (47%)	91 (43%)	
>10-15 cm	23 (5.3%)	17 (7.9%)	
Missing	4 (0.9%)	0 (0%)	
cT-stage*			0.9
T1-T2	17 (3.9%)	10 (4.7%)	
T3	313 (72%)	151 (71%)	
T4	103 (24%)	52 (24%)	
Tx	1 (0.2%)	1 (0.5%)	
cN-stage*			**0.027**
N0	97 (22%)	48 (22%)	
N1	118 (27%)	79 (37%)	
N2	213 (49%)	82 (38%)	
Nx	6 (1.4%)	5 (2.3%)	
cEMVI*			**<0.001**
No	76 (18%)	83 (39%)	
Yes	180 (41%)	81 (38%)	
Missing	178 (41%)	50 (23%)	
Distance to MRF for cT3 tumors			0.8
0-2 mm	195 (45%)	89 (42%)	
>2 mm	105 (24%)	57 (27%)	
Irrelevant (not cT3 tumor)	121 (28%)	63 (29%)	
Missing	13 (3.0%)	5 (2.3%)	
Neoadjuvant radiotherapy			**<0.001**
25Gyx5F	44 (10%)	56 (26%)	
45-52Gyx25-28F	380 (88%)	157 (73%)	
Andet	10 (2.3%)	1 (0.5%)	
Neoadjuvant chemotherapy			**<0.001**
No	54 (12%)	59 (28%)	
Yes	380 (88%)	155 (72%)	
Neoadjuvant chemotherapy type			**<0.001**
5-FU concomitant with radiotherapy	355 (82%)	143 (67%)	
Other combinations of chemotherapy	22 (5.1%)	3 (1.4%)	
None	54 (12%)	59 (28%)	
Missing	3 (0.7%)	9 (4.2%)	
Micro radicality			0.5
No	44 (10%)	26 (12%)	

**Characteristic**	**Missing tumor deposit status**	**Reported tumor deposit status**	**p-value**
Yes	385 (89%)	184 (86%)	
Missing	5 (1.2%)	4 (1.9%)	
Adjuvant chemotherapy			0.7
No	419 (97%)	208 (97%)	
Yes	15 (3.5%)	6 (2.8%)	
Adjuvant chemotherapy type			>0.9
5-FU	6 (1.4%)	2 (0.9%)	
5-FU + oxaliplatin	9 (2.1%)	4 (1.9%)	
None	419 (97%)	208 (97%)	

n (%); mean (minimum to maximum)

5-year distant recurrence probability: Missing: 0.23 (95% CI: 0.19, 0.27), Non-missing: 0.23 (95% CI: 0.18, 0.29)

Figure A: Cumulative incidence of distant recurrence by T-stage

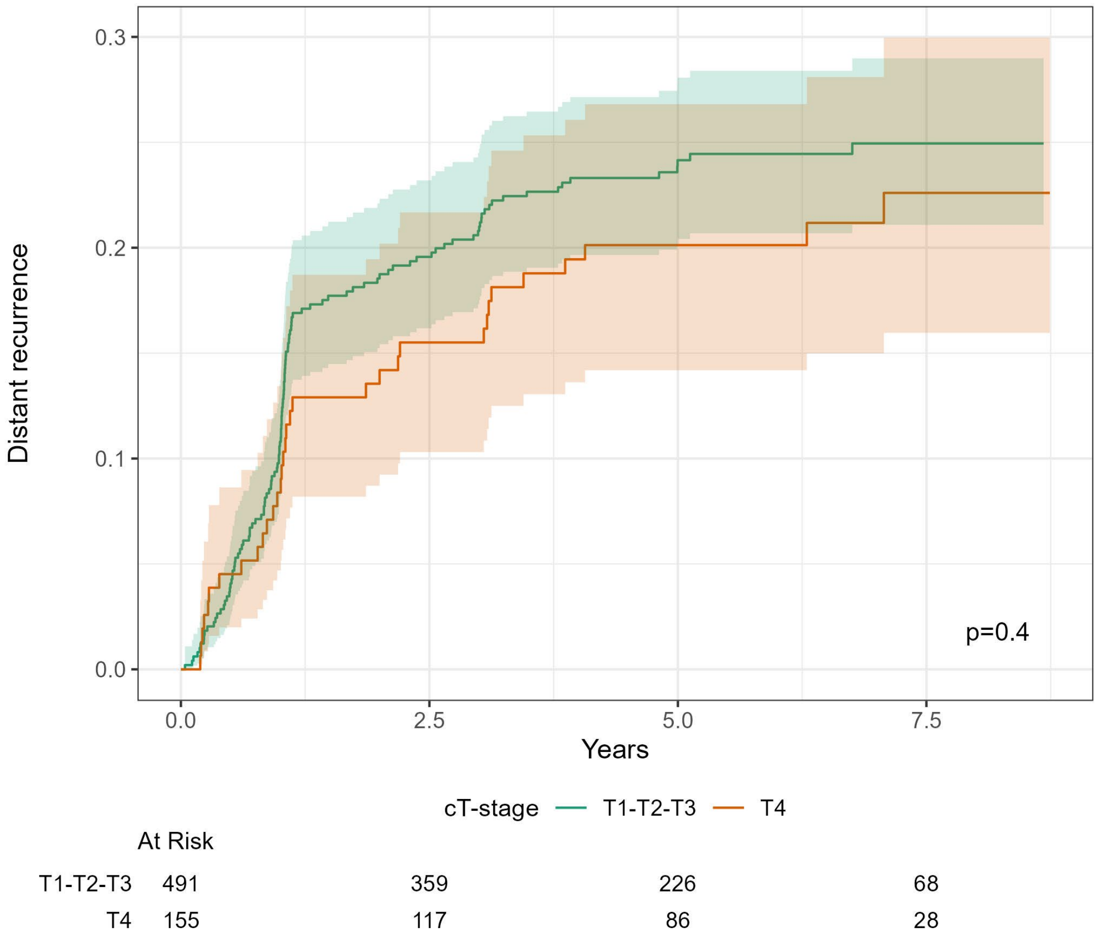

Two patients with cTx-stage documented in the diagnostic MRI report were excluded in analysis.

Figure B: Cumulative incidence of distant recurrence by tumor deposit status

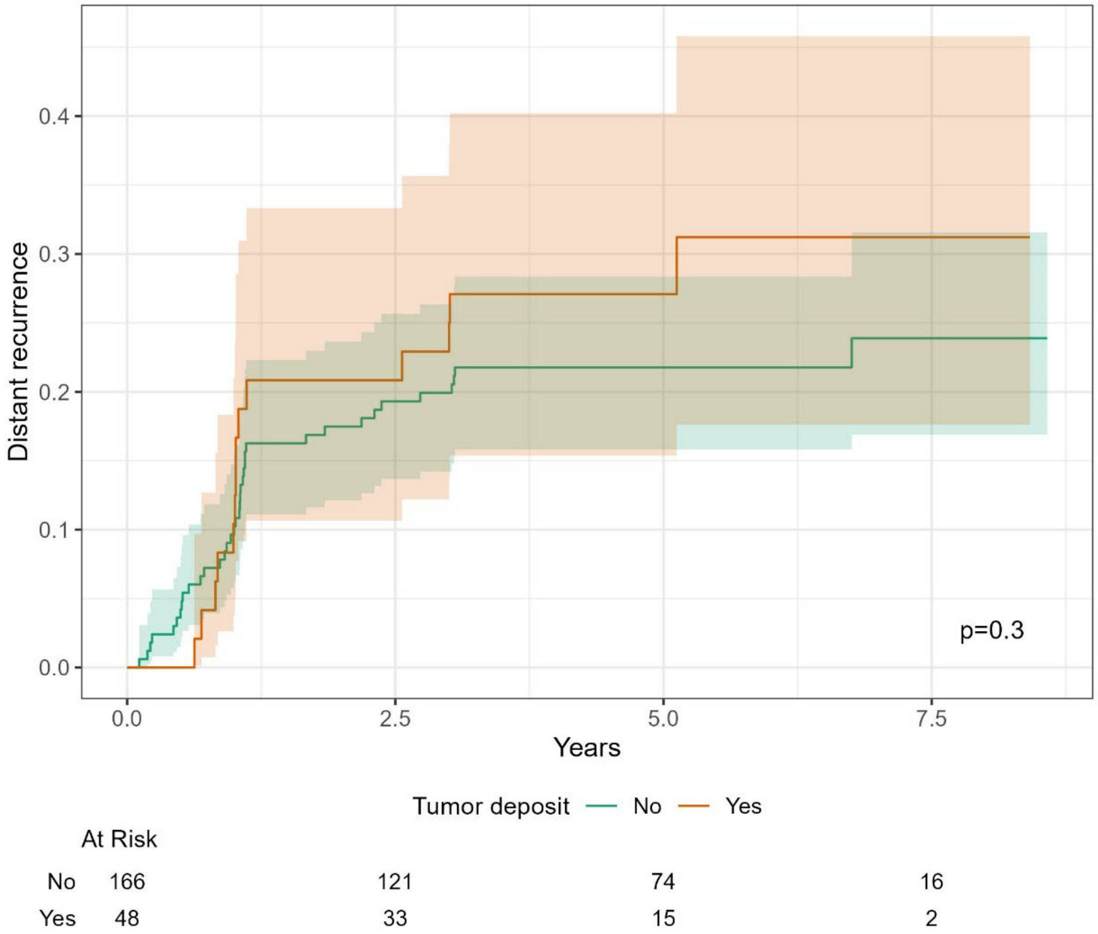

A total of 434 patients were excluded from the analysis due to missing documentation of tumor deposit status in the diagnostic MRI report.

Figure C) Percentage patients with missing cEMVI status in the diagnostic MRI report according to year of diagnosis

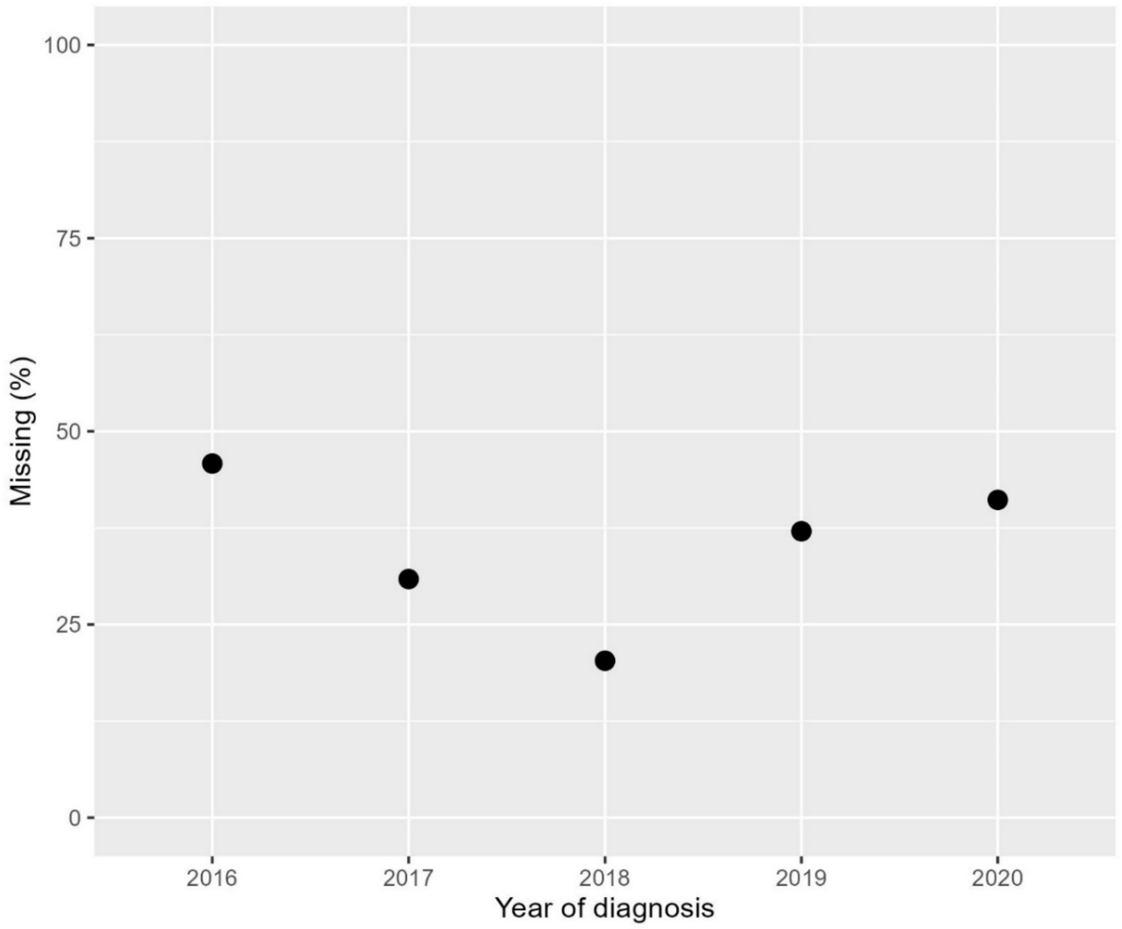

Figure D) Percentage patients with missing tumor deposit status in the diagnostic MRI report according to year of diagnosis

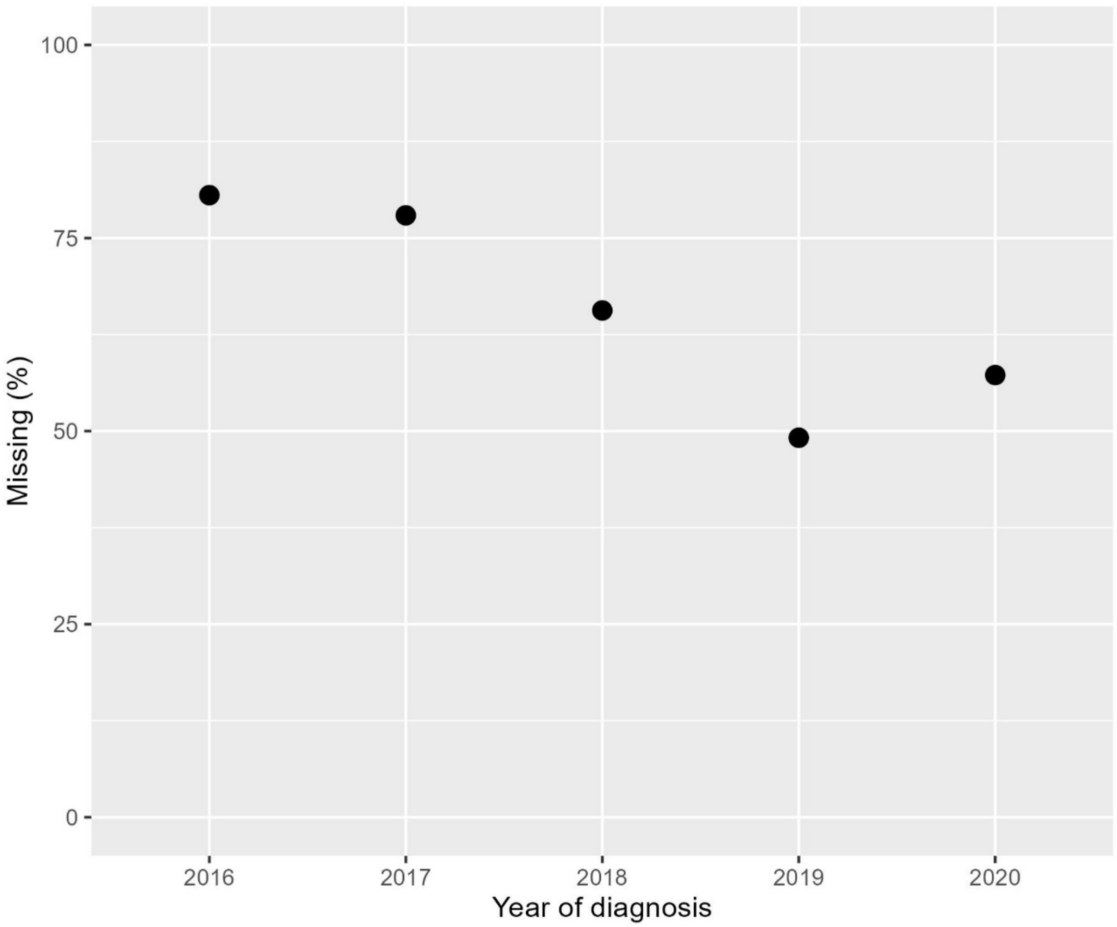

Figure E) Cumulative incidence of distant recurrence by cEMVI status

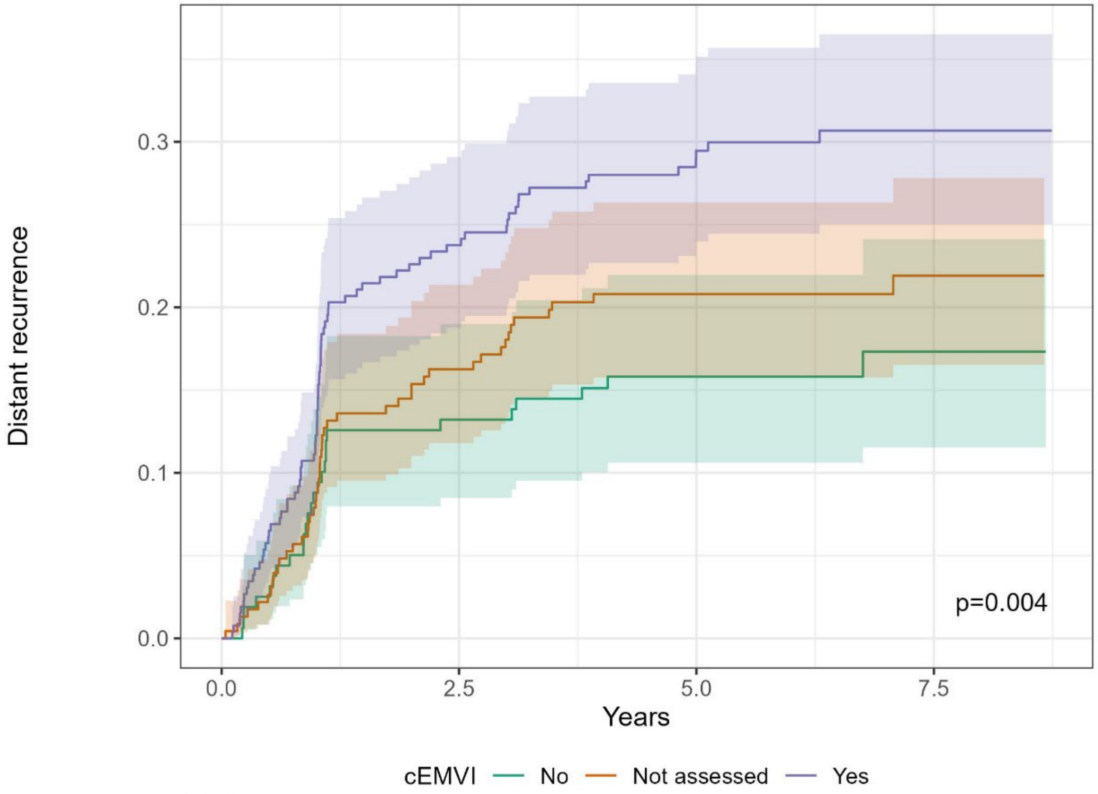

At Risk

No	159	126	90	25
Not assessed	228	173	102	45
Yes	261	179	122	26

cEMVI status for the whole cohort including patients with missing documentation of cEMVI status in the diagnostic MRI report (“not assessed”).
